# Cortical layer-specific differences in stimulus selectivity revealed with high-field fMRI and single-vessel resolution optical imaging of the primary visual cortex

**DOI:** 10.1016/j.neuroimage.2022.118978

**Published:** 2022-02-07

**Authors:** Shinho Cho, Arani Roy, Chao J. Liu, Djaudat Idiyatullin, Wei Zhu, Yi Zhang, Xiao-Hong Zhu, Phillip O’Herron, Austin Leikvoll, Wei Chen, Prakash Kara, Kâmil Uğurbil

**Affiliations:** a Center for Magnetic Resonance Research (CMRR), University of Minnesota, MN 55455, United States; b Department of Radiology, University of Minnesota, MN 55455, United States; c Department of Neuroscience, University of Minnesota, MN 55455, United States; d Department of Neuroscience, Medical University of South Carolina, Charleston, SC 29425, United States

**Keywords:** Functional magnetic resonance imaging, Multi-photon optical imaging, Cerebral blood volume response, Primary visual cortex, Layer-specific orientation selectivity, Vessel dilation

## Abstract

The mammalian neocortex exhibits a stereotypical laminar organization, with feedforward inputs arriving primarily into layer 4, local computations shaping response selectivity in layers 2/3, and outputs to other brain areas emanating via layers 2/3, 5 and 6. It cannot be assumed *a priori* that these signatures of laminar differences in neuronal circuitry are reflected in hemodynamic signals that form the basis of functional magnetic resonance imaging (fMRI). Indeed, optical imaging of single-vessel functional responses has highlighted the potential limits of using vascular signals as surrogates for mapping the selectivity of neural responses. Therefore, before fMRI can be employed as an effective tool for studying critical aspects of laminar processing, validation with single-vessel resolution is needed. The primary visual cortex (V1) in cats, with its precise neuronal functional micro-architecture, offers an ideal model system to examine laminar differences in stimulus selectivity across imaging modalities. Here we used cerebral blood volume weighted (wCBV) fMRI to examine if layer-specific orientation-selective responses could be detected in cat V1. We found orientation preference maps organized tangential to the cortical surface that typically extended across depth in a columnar fashion. We then examined arterial dilation and blood velocity responses to identical visual stimuli by using two- and three-photon optical imaging at single-vessel resolution—which provides a measure of the hemodynamic signals with the highest spatial resolution. Both fMRI and optical imaging revealed a consistent laminar response pattern in which orientation selectivity in cortical layer 4 was significantly lower compared to layer 2/3. This systematic change in selectivity across cortical layers has a clear underpinning in neural circuitry, particularly when comparing layer 4 to other cortical layers.

## 1. Introduction

The fidelity of mapping hemodynamic signals mediated by neurovascular coupling onto neural activity is a critically important question that impacts numerous neuroimaging methods employed to study the brain. For relatively coarse spatial scales of neural representations across the neocortex, hemodynamic signals almost always track changes in aspects of neural activity (synaptic or spiking). These include, among many examples, resting state (He et al., 2018), and feature-selective responses such as tracking the location and preferred orientation of a visual stimulus (e.g. O’Herron et al., 2016; Shen et al., 2012; Shmuel and Grinvald, 1996; Yacoub et al., 2008). However, individual functional patches of neocortex are not point processors. Instead each patch has a rich laminar organization of neural circuitry that results in predictable changes in neural selectivity across cortical layers. Specifically for orientation selectivity in the primary visual cortex (V1), single-neuron responses from rodent and non-rodent mammals show that orientation selectivity changes systematically across cortical layers (O’Herron et al., 2020; Ringach et al., 2002; Van Hooser et al., 2013). The most recent of these studies attributes the laminar differences in selectivity to the location of thalamic inputs being largely confined to cortical layer 4 and these thalamic inputs having very weak orientation selectivity (Chung and Ferster, 1998; Ferster et al., 1996; Kara et al., 2002; O’Herron et al., 2020).

The neural representation of orientation selectivity across cortical layers in V1 provides an ideal test for the fidelity with which hemo-dynamic signals map onto neural signals across cortical layers. That is not to say that they will be identical, because hemodynamic signals can spread over larger cortical regions compared to neural activity (e.g. Duong et al., 2001; O’Herron et al., 2016). Instead, the presumption is that differences in neural selectivity across cortical layers may be large enough to be reflected in differences in hemodynamic signals, assuming that the spatial resolution of the hemodynamic measurement is sufficient. The highest resolution hemodynamic signal is that which comes from a single blood vessel, e.g., dilation and/or blood flow change (He et al., 2018; O’Herron et al., 2016; Shen et al., 2012). Regardless of the angioarchitecture of the neocortex (Blinder et al., 2013; Kirst et al., 2020; Weber et al., 2008), if an imaging technique, such as variants of functional magnetic resonance imaging (fMRI) or intrinsic signal optical imaging, matches single-vessel hemodynamic responses at a specific voxel resolution, then at that resolution or better, it can be considered to have the capability to examine and capture laminar-specific differences in stimulus selectivity.

Past fMRI studies from both cat and human V1 did not have the spatial resolution to track laminar differences in orientation selectivity (Duong et al., 2001; Fukuda et al., 2006; Yacoub et al., 2008). Similarly, optical imaging techniques such as intrinsic signal or two-photon imaging could only focus on the most superficial cortical layers in V1 (Bonhoeffer and Grinvald, 1993; O’Herron et al., 2016; Shmuel and Grinvald, 1996; Swindale et al., 2003). With techniques like fMRI, there is also the question of how accurately the hemodynamic signal alterations are reflected in the fMRI functional maps because they are derived indirectly through complex mechanisms that can impact the magnitude and/or the spatial extent of the hemodynamic responses mediated by neurovascular coupling (e.g. Ugurbil, 2016).

Here we take advantage of the well-known neuronal functional micro-architecture of cat V1 to determine if the sharpness of orientation tuning across cortical layers can be detected with two different types of hemodynamic signals: cerebral blood volume (CBV) weighted fMRI and multi-photon (two- and three-photon) imaging of dilation and blood velocity in single blood vessels.

## 2. Materials and methods

### 2.1. Surgery and anesthesia

All fMRI experiments were performed at the University of Minnesota whereas optical imaging experiments were performed both at the Medical University of South Carolina and the University of Minnesota. All surgical and experimental procedures used in each imaging modality were approved by the respective Institutional Animal Care and Use Committee at each institution and were entirely consistent with the National Institutes of Health (NIH) Guide for the Care and Use of Laboratory Animals.

Thirty-two cats of both sexes were used (7 for fMRI and 25 for optical imaging). They were anaesthetized with isoflurane (1–2% during surgery, 0.5–1.1% during imaging) and paralyzed with a continuous intravenous infusion of vecuronium bromide (0.2–0.3 mg kg^–1^ h^–1^ ) (O’Herron et al., 2016). Cats were artificially ventilated and the end tidal CO_2_ was regulated at 3.5–4.5%. Heart rate, respiration rate, and temperature were also monitored. In optical imaging experiments, an electroen-cephalogram was also used. Moreover, for optical imaging, craniotomies were made over area 18 of V1 (Kara and Boyd, 2009; O’Herron et al., 2012; Payne, 1990), the dura was reflected, and the craniotomies were sealed with 3% agarose and a glass coverslip (O’Herron et al., 2012).

### 2.2. Visual stimuli for fMRI experiments

Visual stimuli were drifting square-wave gratings (100% contrast; drifting in 2 Hz) presented on a projector viewed 18 cm from the eyes of the cat positioned in the scanner bore. The stimuli were presented at 16 directions of motion (8 orientations) in 22.5° steps, in random order. Each grating stimulus was displayed for 5 or 10 s followed by 20 s of blank (equiluminant gray screen). Each condition was repeated 22–26 times.

### 2.3. Visual stimuli for optical imaging experiments

For two- and three-photon imaging, drifting square-wave grating stimuli (100% contrast, 2 Hz temporal frequency) were presented on a 17-inch LCD monitor placed 30 cm from the eye. The stimuli were presented at 8 directions of motion in 45° steps, in pseudo-random order. Each grating stimulus was displayed for approximately 5 s with about 20 s of blank (equiluminant gray screen). The duration of the stimulation period and the duration of the blank period were always identical across all epochs in a stimulus sequence. Each condition was repeated 3–6 times.

### 2.4. MR imaging

All data were collected using a 9.4T/31 cm horizontal MR system with an actively shielded gradient coil (400 mT/m, a rise time: 120 *µ*s) (Varian Inc.). A custom-built proton radio frequency (RF) coil (single channel, single 15-mm diameter round loop) was positioned on the sagittal crest of the skull near the visual cortex.

The structural images were obtained with following image sequence and parameters: flow-compensated, 2D RF-spoiled, gradient-echo (GRE) sequence; matrix size = 256×256, field of view (FOV) = 32×32 mm, inplane resolution = 125×125 *µ*m, slice thickness = 125 *µ*m, 10–12 axial slices, repetition time (TR) = 119.9 ms, echo time (TE) = 5.1 ms.

For functional imaging an exogenous contrast agent (Feraheme®, 10–20 mg kg^–1^ ) was infused intravenously. Then, the following fMRI pulse sequence and parameters were used. 7 axial or 3 sagittal slices with segmented, multi-shot (*number segments* = 4), 2D gradient-recalled-echo (GRE), echo-planar-imaging (EPI) with center-out phase-encoding (Kim et al., 1996); matrix size = 80×80 (axial), 128×128 (sagittal), slice thickness = 250 *µ*m; 6–8 slices for axial and 1–3 slices for sagittal/coronal imaging; FOV = 20×20 mm (axial), 32×32 mm (sagittal); in-plane resolution = 250 × 250 *µ*m; volume TR = 2000 ms; TE = 10 ms; repetition number = 130; total acquisition time of 260 s per fMRI run.

### 2.5. MR data preprocessing and analysis

The Analysis of Functional Neuroimages (AFNI) (Cox, 1996) was used to pre-process fMRI data in the following order: spike removal, slice-timing correction, motion correction (6 Degrees of Freedom; 3 translations and 3 rotations), baseline drift removal with six-order polynomial modeling, and spatial smoothing (250 *µ*m, full-width-half-maximum Gaussian-kernel). The smoothing broadened the point-spread-function (PSF) of the wCBV images by approximately 1.5 voxels to 360 *µ*m, but did not have a perceptible impact on the estimate of depth-dependent CBV response. All individual images before and after pre-processing were inspected visually. General linear modeling was used to estimate and map the CBV responses to each different orientation stimulus (3dDeconvolve, AFNI software). Previously known impulse response model of iron oxide-based contrast agent was used with deconvolving parameters (stimulation duration and stimulus on-set time) (Leite et al., 2002) and outputs of the model were beta coefficient, correlation coefficient (CC), and F-statistics. To generate wCBV activation maps, we used two statistical thresholds in sequence. First, we applied a threshold of voxel-wise F-statistic (*F* = 4.0, *P* = 0.001) to the general linear modeling (GLM) output. Then we applied the cluster-size-based threshold (minimum cluster size > 40 voxels with two-sided second-nearest neighbor clustering; 3dClustSim, AFNI software) to remove the voxel clusters that were defined as the false-positive response (False Discovery Rate [FDR] corrected at *q* = 0.05). As a result, 644 false-positive clusters among 871 clusters were masked out, mostly from outside of the cortical region.

Apparent activation from large pial veins was suppressed using a threshold on the logarithm of the standard deviation of the time series divided by its mean (log(Std/Mean)) eliminating voxels with log(Std/Mean) > 1.6 (see Fig. S1 and Chen et al., 1999; Kim et al., 1994; Zhao et al., 2006). The parameter (Std/Mean) is the inverse of temporal SNR (tSNR), therefore, this procedure eliminates voxels with very low tSNR. Finally images of stimulus-induced signal changes representing functional maps were overlaid on individual structural image with appropriate color-encoding and visualized with ITK-snap software (Yushkevich et al., 2016). An anatomical 3D model of the cortex was rendered based on cat brain atlas (Stolzberg et al., 2017) with graphics software (Blender®).

### 2.6. Cortical flattening for MR data

**Fig. 1.**
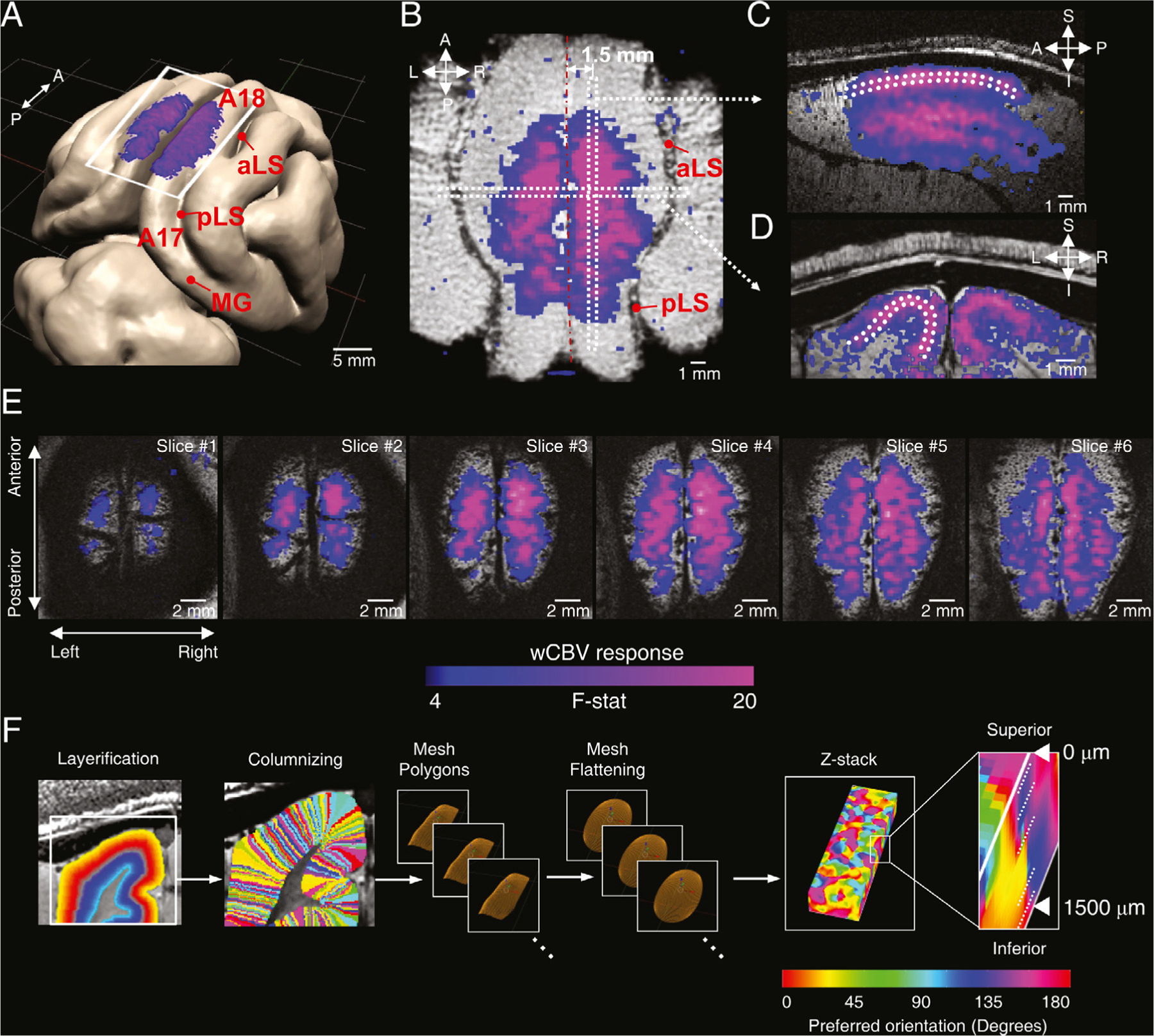
CBV-weighted (wCBV) fMRI signal changes across the cortex evoked by visual stimuli. Blue-purple colors represent the F-statistics of general linear model (GLM) fitting to cross-trial averaged responses across all 8 stimulus orientations (F-stats > 4.0, *P* < 0.001, cluster-extent based false discovery rate corrected at *q* = 0.5). (A) Area 18 of cat V1 approximately delineated by the white rectangle on a 3D rendered cat brain model, with the stimulus-evoked signal changes observed on the cortical surface. (B–D) The stimulus-evoked wCBV changes observed in the axial (B), sagittal (C), and coronal (D) planes (acquired separately using axial, sagittal and coronal imaging slices) are shown overlaid on corresponding GRE anatomic images. The white-dotted line shows the experimentally determined upper and lower boundary of cortical layer 4 (see Fig. 2C). (E) Activation maps for six contiguous 250 *μ*m thickness axial slices. (F) This shows the procedure of cortical flattening for laminar analysis. The cortical boundary between CSF and GM, GM and WM was defined, then ‘layerification’ and ‘columnizing’ was carried on the LAYNII software, (Huber et al., 2021). The depth and column labeled voxels were reconstructed into multiple equal-depth 2D planes (‘flattening’) by the mesh parameterization (Peyre, 2021). The in-plane orientation preference was estimated, and the planes were stacked up along with cortical depth (0 – 1500 *μ*m) (right most panel in Fig. 1F) (see the Materials and Methods for details) Abbreviations in panel A: aLS, anterior lateral sulcus; CSF, cerebrospinal fluid; GM, gray matter; MG, marginal gyrus; pLS, posterior lateral sulcus; WM, white matter.

Due to the curvature of the cortex, the axial slice orientation employed in data acquisition does not match actual cortical layers. Therefore, the fMRI data were interpolated, then flattened and individual voxels were remapped to a 3D grid, in which the equal-depth voxels were aligned in the same 2D plane; this ‘layerification’ process produced images of 2D planes with a constant depth interval from the cortical surface (spacing between planes 62.5 *µ*m) (see Fig. 1F).

Cortical flattening consists of multiple steps with combination of different software adopted for following procedure: cortical segmentation (ITK-snap, Yushkevich et al., 2016), layer and column labeling (‘layerification’ and ‘columnizing’) (LAYNII, Huber et al., 2021), cortical flattening (LAYNII, Huber et al., 2021), 3D reconstruction (MATLAB R2018a, MathWorks Inc.), and visualization (ITK-snap and MATLAB). First, high-resolution anatomical images for each animal (GRE image, 125 *µ*m isotropic voxel) were interpolated by bi-cubic interpolation (interpolated to 62.5 *µ*m isotropic voxel), where anatomical images were co-registered to the mean EPI image. Second, the cortical boundary between cerebral spinal fluid (CSF) and gray matter (GM), as well as GM and white matter (WM) were defined semi-automatically based on EPI image contrast and intensity cut-off threshold. Once boundaries were defined for each slice, a custom-built MATLAB program was applied for surface fitting in 3D (cubic spline) in order to obtain a smooth, continuous surface mesh in 3D space (MATLAB R2018a, MathWorks Inc.). Third, LAYNII software carried out ‘layerification’ and ‘columnizing’ for voxels between the CSF-GM and GM-WM boundaries, in which the delineated boundaries also divide the cortex into multiple image layers with ‘growing layer’ and equal-distance approach (see details in (Huber et al., 2021) and https://layerfmri.com/). At this stage, cortical depth and anatomical columnar membership of each voxel was determined. Fourth, the coordinate information (X, Y, and Z) of labeled voxels was converted to a 3D mesh data structure, and then the mesh parameterization was carried out for “remapping” of the equal-depth voxel groups to the same 2D plane (Toolbox Graph, Peyre, 2021). This process was repeated for all cortical depths up to 40 contiguous equal-depth planes (62.5 *µ*m spacing along with depth axis). During the process, the coordinate transformation matrix for voxels was obtained. Finally, the transformation matrix was applied to remapping EPI voxels, yielding GRE EPI voxels that were aligned in equal-depth 2D planes across all three dimensions, such that the location of voxels corresponds to the flattened anatomical image volume. Visual inspection was carried out for each flattened 2D layer before being stacked to form a 3D volume (Huber et al., 2021; Kemper et al., 2018).

### 2.7. Estimation of column-wise angular deviation

Following the cortical flattening, the columnar angular deviation was calculated for each animal (*n* = 4) and averaged (Fig. S7). This calculation was performed for each vertically stacked voxel that span the cortical layers (depth: 0∼1500 um), perpendicular to the cortical surface, i.e., voxel column (Fig. S7A left panel). Specifically, we estimated the column-wise mean of orientation preference by averaging voxels’ reference affiliated in the same column. Then circular, absolute angle distance between individual voxels’ preferred orientation and columnar mean was averaged over all voxel-columns, subsequently being applied in the seven caudal-rostral slices for each cat and finally group average was obtained (Fig. S7B–C). Additionally, to further validate the integrity of the orientation map, we shuffled the location of voxels in the sagittal plane and calculated the angular deviation identically to the way it was done to obtain the normal orientation map (see Fig. S7A right panel).

### 2.8. Multi-photon imaging

Two- and three-photon optical imaging was performed with a customized microscope from Bruker with two separate excitation sources for two- and three-photon imaging, as described previously (Liu et al., 2020). Imaging was performed using a 25× objective lens (XLPLN25XWMP2, NA 1.05, Olympus). For imaging dilation from arteries and blood velocity from arteries and capillaries, aqueous solutions of fluorescent dyes were injected intravenously. In the present study, we did not examine veins for visually-evoked responses. In our previous work, we demonstrated that veins do not show visually-evoked dilation to drifting grating stimuli (Shen et al., 2012). Arteries were identified based on their elastin wall label with Alexa Fluor 633 hydrazide and/or visual inspection of the direction of blood flow after following pial arteries diving into the parenchyma (O’Herron et al., 2016; Shen et al., 2012). The nomenclature of identifying capillaries is controversial whereby some groups identify pre-capillary arterioles with smooth muscle as capillaries and other groups identify capillaries where only single-file red blood cell flow is possible and where the vessel walls are likely devoid of smooth muscle, i.e., capillaries that have vessel diameters of 4–7 *µ*m (Damisah et al., 2017; Drew et al., 2011; Hall et al., 2014; Hill et al., 2015; Hillman et al., 2007; Kleinfeld et al., 1998). Moreover, the transition zone between pre-capillary arterioles and these 4–7 *µ*m diameter capillaries is, to date, anatomically undefined (Hartmann et al., 2021). In our past and the current work, we define capillaries as vessels with a diameter of 4–7 *µ*m and wherein we can reliably assess blood velocity changes when using visual stimulation. Blood vessels in layer 2/3 were imaged in the two-photon mode using one of three fluorescent dyes: Alexa Fluor 633 hydrazide (excitation 810 nm), Texas Red dextran (70 kD; excitation 920 nm) or fluorescein dextran (2000 kD; excitation 810 nm). Layer 4 vessels were imaged either in the two-photon mode using Texas Red dextran (70 kD; excitation 1100 nm) or the far red-shifted Alexa 680 dextran (2000 kD; excitation 1240 nm), or in the three-photon mode using fluorescein dextran (2000 kD; excitation 1300 nm). The emitted photons were separated into green and red channels by first passing through a dichroic beam splitter (T565lpxr, Chroma) and then through barrier filters (green: 525 ± 25 nm bandpass, Chroma; red: 675 ± 75 nm bandpass, Chroma), before being collected by two photomultiplier tube (PMT) detectors (H10770PB-40 SEL, Hamamatsu).

### 2.9. Quantifying blood vessel dilation in optical imaging data

Blood vessel dilation data were analysed as described previously (O’Herron et al., 2016; Shen et al., 2012). Briefly, penetrating arterioles were visualized either through labeling of the vessel walls with Alexa Fluor 633 hydrazide or through labeling of the lumen with dyes such as fluorescein dextran, Texas Red dextran or Alexa 680 dextran. Vessel diameter was determined in full-frame images by one of two methods. When the vessel had a circular profile, a region of interest was manually drawn around each vessel and a circle was fit to the pixels in the region that passed a luminance threshold. For vessels with an elongated profile, a cross-section was taken through the vessel walls and the peaks in luminance (for the wall labeling) or peaks in the pixel-by-pixel luminance difference along the line (for lumen labeling) were used to compute the diameter. To estimate the vascular response to each stimulus orientation, a response window was defined and the mean response across this time window was divided by the baseline level to get the percentage change in diameter. Responsive vessels were defined by ANOVA across baseline and eight stimulus directions over multiple trials (*P* < 0.05).

### 2.10. Quantifying blood velocity

Velocity data were analyzed as described previously (Chhatbar and Kara, 2013; O’Herron et al., 2016; Shen et al., 2012). Briefly, line scans were first pooled into blocks of 250, 500, 1000 or 2000 lines. The angle of the RBC streaks in each image was used to determine the velocity of that block and a time course of velocity measurements was extracted. Baseline and stimulus windows were defined similarly to the dilation analysis and equivalent OSI and statistical analyses were performed.

### 2.11. Orientation preference mapping for fMRI data

The preferred orientation of a voxel was estimated by fitting tuning curves to 8 orientation responses and topographically mapped on 3D space with differing colors. All curve fitting was conducted with voxels from flattened cortex in each hemisphere. For each voxel, all trials for a given orientation were averaged. The curve was fit to those averaged responses with the von Mises function, yielding four fitting parameters determined by following formula (Swindale, 1998):

$$O\left(\text{θ}\right)=A\cdot e^{k\left(\cos2\left(\theta -\theta_{p}\right)-1\right)}+B$$

where *O* is the model response, theta (θ) is an independent variable representing the orientation of the stimulus, *θ_p_* represents the preferred orientation for a given tuning curve, and kappa (*k*) is a width parameter of the tuning curve. A and B are the maximum height and intercept of the tuning curve. The initial value for *θ_p_* was randomly chosen between 0° and 180° for every fitting trial in order to minimize the estimation bias to determine the preferred orientation. Note that no prior normalization was conducted for input wCBV responses to minimize the chance for fitting local maxima. The following formula was further applied to estimate the preferred orientation *θ_p_* in order to unwrap the phase:

$$\theta_{p}={\left|\text{O}\left(\theta \right)\right|}_{mod\;\;180\cdot }$$

The fit was rejected, and the corresponding voxels were eliminated from further analysis, if any of the following constraints were met: i) convergence not occurring after 300 iterations of fitting; (ii) the goodness of fit *R*^2^ < 0.2; (iii) if the peak amplitude, *A* < - 20 or *A* > *20.* R-square statistics of individual animals obtained across all cortical depths and each orientation condition are presented as frequency histogram and line plot (Fig. S5).

For visualization, the preferred orientation of voxels was encoded with a Hue Saturation Value (HSV) color scale; where red color indicates the orientation preference corresponding to 0° or 180° All analysis was performed in MATLAB (MathWorks, Inc.) and visualization was performed by ITK-snap (Yushkevich et al., 2016) and custom code.

The orientation tuning selectivity was calculated from each voxel, equal-depth aligned for cortical depth in the flattened hemisphere. We measured the wCBV responses for 8-orientation conditions and calculated the orientation selectivity index (OSI) as 1 – Circular Variance:

$$\text{OSI}=\text{abs}\left[\frac{\sum_{k}r_{k}e^{i2\theta_{k}}}{\sum_{k}r_{k}}\right]$$

where *r_k_* is the trial averaged wCBV response (-Δs∕s) of each orientation, in which each orientation condition was repeated up to 26 times in an experiment. Additionally, for the OSI calculation, voxels were excluded 1) if those did not meet the aforementioned selection criteria in tuning curve fitting, and 2) if the mean wCBV response was lower than 1%, which is to minimize the OSI bias by poorly responding voxels. OSI values of voxels positioned in the same cortical depth on the interpolated grid were averaged and plotted for each animal and the group average (e.g., see Fig. 4A). The same OSI calculation was performed for optical imaging results. To compare the layer-dependent OSI between fMRI and optical imaging, OSI values that fell into two depth windows 160–410 *µ*m (for layer 2/3) and 650–910 *µ*m (for layer 4) were compared (see Fig. 6).

### 2.12. Identifying cortical layer 4 in histology

The VGLUT2 antibody labels the end terminals of thalamic afferents in cortical layer 4 (Nahmani and Erisir, 2005; Nakamura et al., 2007). We therefore used this approach to determinate the depth of the upper and lower boundary of layer 4 in cat area 18 in postmortem histological sections. These anatomical boundaries were then used to assign functional data collected at different cortical depths to different cortical laminae. To collect histological sections, animals used for imaging were euthanized by intravenous injection of barbiturate (pentobarbital 195 mg kg^–1^ , phenytoin sodium 25 mg kg^–1^ ) and transcardially perfused with phosphate buffered saline followed by 4% paraformaldehyde. The brains were removed and 50 *µ*m thick coronal sections of area 18 of V1 were cut on a vibratome. Two VGLUT2 antibodies from different vendors were used individually (one type of antibody per section) with equivalent results (see Fig. S9). When using the primary VGLUT2 antibody from Millipore (guinea pig AB2251-I), for fluorescence detection we used the secondary antibody goat anti-guinea pig IgG Alexa 594 (Abcam AB150188). When using the other primary VGLUT2 antibody (Synaptic Systems, rabbit 135,403), for fluorescence detection we used the secondary antibody goat anti-rabbit IgG Alexa 594 (Invitrogen A11037). The general labeling protocol was as follows. Tissue sections were incubated overnight in the primary antibody solution (1:1000 dilution and 2% Triton X-100, prepared in 10% goat serum) at 4 °C. Following three PBS washes for 15 min each, the sections were incubated for 2 h in the secondary antibody solution (1:750 dilution and 2% Triton X-100, prepared in 10% goat serum) at room temperature. Following three PBS washes for 15 min each, the stained sections were then mounted on glass slides, dried, and covered using a coverslip with Fluoromount aqueous mounting medium (Sigma). The sections were imaged on a confocal microscope (Nikon A1R) and the fluorescent images of VGLUT2-stained sections were processed in ImageJ to measure line intensity profiles along the depth of the cortical sections.

## 3. Results

### 3.1. Spatial extent of CBV activation in V1 in response to visual stimulation

We used multi-slice T_1_-weighted GRE (gradient-recalled-echo) and T_2_*-weighted, multi-segment, 2D GRE Echo Planar Imaging (EPI) sequences to obtain anatomical and functional images, respectively, in area 18 of V1. CBV weighted functional imaging (wCBV fMRI) with 250 *µ*m isotropic acquisition resolution was performed using an exogenous contrast agent and a block design paradigm to map visually evoked responses to drifting oriented gratings (8 different orientations). Data included in the analysis were obtained from 7 contiguous axial slices in 4 cats, and 3 sagittal slices in 3 cats. In one of these cats, functional maps were acquired in axial, sagittal and coronal orientation for visualization purposes (Fig. 1).

Drifting grating stimuli evoked robust responses across the entire dorsal surface of area 18 of the cat primary visual cortex (Fig. 1A,B) and also along the medial bank (Fig. 1D). This spatial extent was further evident in functional activation maps in contiguous axial slices (Fig. 1E). In generating these images, activation originating from large pial veins in the interhemispheric fissure was suppressed in post-processing by applying an upper-limit cut-off threshold for signal fluctuations in the fMRI time series (Chen et al., 1999; Kim et al., 1994) (Fig. S1). Across the cortical layers within area 18, which is the region of interest in our study, the stimulus-induced signals appeared to first increase along the cortical depth and then decrease after passing through the middle layer of the visual cortex (see Fig. 1D). These CBV activation results are consistent with previous fMRI studies in cat V1 (Fukuda et al., 2006; Zhao et al., 2005).

We next quantified the change in CBV signal across the depth of the cortex in area 18, and examined its potential relationship to known features of cortical anatomy, e.g., the precise location of layer 4.

### 3.2. Quantifying cortical layer-dependent CBV-weighted signal changes to visual stimulation

Because of the cortical curvature, quantifying stimulus-evoked signals across cortical layers required converting images acquired with contiguous axial slices to equal-depth-aligned voxels representing planes parallel to the cortical surface (“cortical flattening”) (Huber et al., 2021) (see Methods, and Fig. 1F). The goodness of the cortical flattening algorithm can be assessed by applying it to T2* weighted, high resolution GRE anatomic images where intracortical veins that run perpendicular to the surface appear as dark lines; maintaining their perpendicular relationship to the cortical surface implies that in the flattened cortex image, they should run vertically as they do (Fig. S2).

**Fig. 2.**
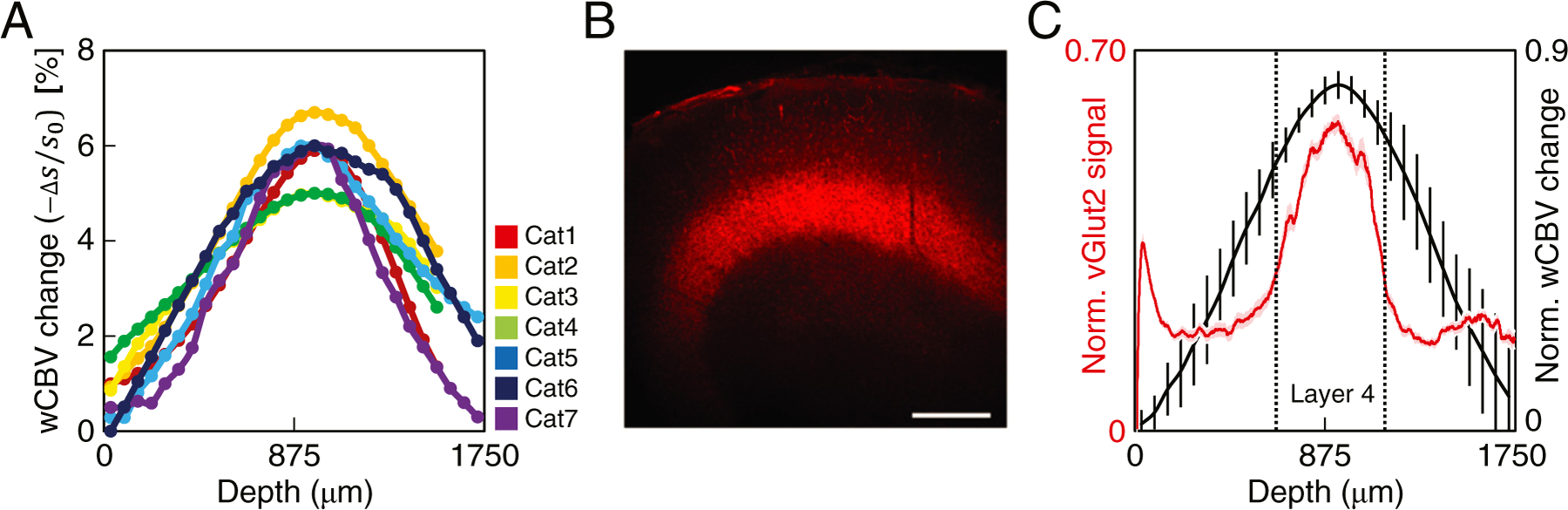
Depth-dependent wCBV fMRI responses across anatomically-validated cortical layers. (A) The stimulus-induced wCBV signal changes across cortical depth from different animals; *n* = 7 cats, 4 studied with axial slices (Cat 1–4) and 3 with sagittal slices (Cat 5–7). The measurements on the data acquired with axial slices were derived from the flattened, layerified cortex (Fig. 1F). (B) A coronal section from cat area 18, immuno-stained for VGLUT2, which labels the end terminals of thalamic afferents in cortical layer 4. A brightness profile was measured over straight lines orthogonal to the cortical surface of the brain. Scale bar 500 *μ*m. (C) Black line represents the group averaged wCBV fMRI response from the individual cases (Cat 1–7) shown in A (error bars denote standard error of the mean, SEM). Red line is the depth-dependent VGLUT2 signal along the cortical depth, averaged across all tissue sections and animals. The pink error band represents the SEM of the normalized fluorescence. Dotted vertical lines represent the upper (650 *μ*m) and lower (1150 *μ*m) boundaries of layer 4 as determined from our histological data.

The high voxel resolution used for fMRI along with cortical flattening and statistical techniques (see Methods) enabled us to determine the stimulus-evoked changes in wCBV fMRI signals across cortical layers. We found that the visual stimuli evoked small CBV changes near the cortical surface, then progressively increased to peak at ∼6% at a cortical depth of 950 *µ*m, before decreasing with increasing cortical depth (Figs. 2A–C, and S3). To determine how this CBV activation profile registers with the laminar anatomy of the visual cortex, the dorsal and ventral boundaries of cortical layer 4 were estimated by immuno-staining of brain sections obtained from visual cortical area 18 for the axonal terminal protein VGLUT2 (Fig. 2B). The intensity of the VGLUT2 staining was measured along straight lines drawn orthogonal to the cortical surface (*n* = 24 lines in 6 brain sections from 2 cats). The VGLUT2 signal reached 25% of the peak brightness at 650 *µ*m and 1150 *µ*m, which we defined as the upper and lower boundaries of layer 4, respectively (Fig. 2C). We found that the peak wCBV signal change coincided with the peak VG-LUT2 intensity (Fig. 2C, compare black and red lines), thereby implying that the stimulus-induced wCBV signal change peaked in the middle of cortical layer 4.

### 3.3. Columnar arrangement of orientation preference

Key features of the lateral organization of the cortical orientation map tangent to the cortical surface, such as orientation domains around pinwheel singularities (Fig. S4) and the spacing of pinwheels were replicated from our flattened volumetric fMRI data.

**Fig. 3.**
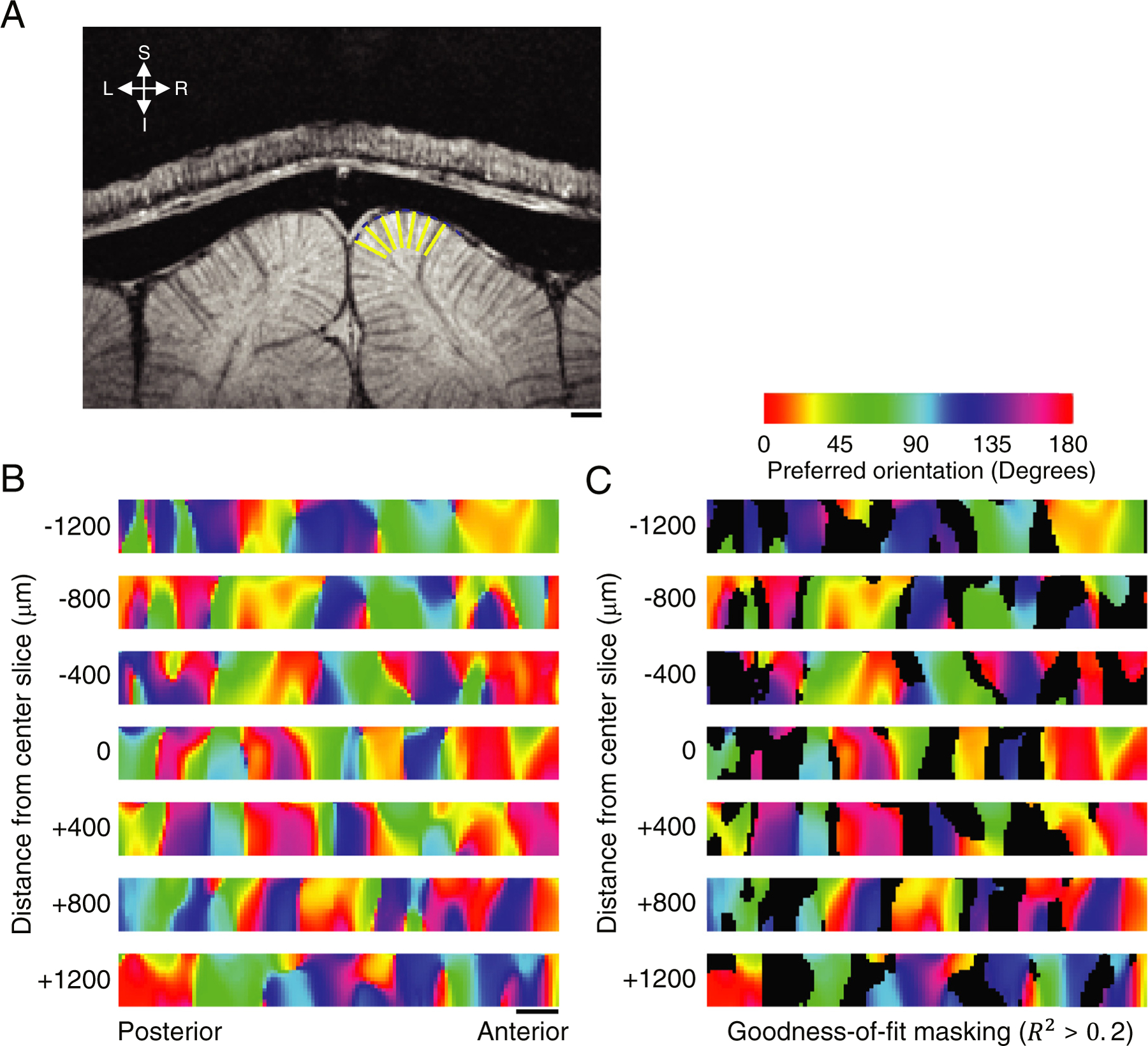
Columnar structure of iso-orientation domains using wCBV fMRI. The flattened volume data derived from the images obtained with axial slices were employed to extract planes perpendicular to the cortical surface running in the rostral-caudal direction. Data from an exemplar cat is illustrated. (A) An anatomical image of a coronal slice acquired before the exogenous contrast agent injection, showing dark lines running within the cortex and perpendicular to the cortical surface arising from penetrating (radial) intracortical veins; the yellow lines on the right hemisphere indicate the locations of planes-of-interest as they intersect this anatomical coronal image perpendicularly to the plane of this image. The orientation of these yellow lines are also perpendicular to the cortical surface. (B) Orientation preference maps observed on the seven planes corresponding to the yellow lines shown in (A) (400 *µ*m equally distanced in the flattened volume); each color map depicts the orientation preference along the cortical depth (vertical dimension) vs. the horizontal axis representing the distance along the rostro-caudal direction. Scale bars indicate 1 mm; (C) Orientation preference map as in panel B, but after excluding pixels (black) that had a goodness-of-fit statistics that was lower than R^2^ < 0.2 (*F* < 13.01 and *P* > 1.0 ×10^−7^ ).

The high spatial resolution of these data permitted the calculation of these features as a function of cortical depth (Fig. 3) and enabled the examination of the laminar organization of preferred orientation and selectivity. Orientation preference maps obtained from the flattened, volumetric functional data were extracted for seven planes that ran perpendicularly to the cortical surface in the rostro-caudal direction in area 18; an example from one animal is shown in Fig. 3. These maps display the laminar pattern of orientation preference along the rostro-caudal dimension.

Typically, we found that the orientation preference from wCBV fMRI was preserved across cortical layers, showing a largely columnar organization extending from the surface to deeper cortical layers. More specifically, the orientation columns appeared perpendicular to the cortical surface; however, some columns were either tilted or fractured in the middle. These features are not likely to have resulted from poor fitting because they were still preserved even after excluding voxels with lower goodness-of-fit statistics (R^2^ < 0.2) (Fig. 3C). Moreover, the R^2^ was similar across cortical depth (R^2^ = 0.31 ± 0.07, mean ±1 SEM, *n* = 7 cats) (Fig. S5).

Subsequent to contrast agent infusion, both intracortical penetrating (arterial) and emerging (venous) blood vessels, which run perpendicular to the cortical surface, are expected to have low signal intensity and SNR in their vicinity. This arises from the shortened T_2_* near the blood vessels caused by the difference in magnetic susceptibility between contrast-agent containing vessel interior and the surrounding tissue. Consequently, such intracortical blood vessels can be observable as approximately vertical lines or bands of low signal intensity in the post-contrast EPI images of the flattened cortex (Fig. S6) from which the orientation preference maps of Fig. 3B and C are derived. The low SNR associated with these blood vessels coupled with the higher temporal fluctuations they may display can lead to poor detectability of orientation preference in their vicinity. Although the resolution of these EPI images is not sufficient to capture the intracortical blood vessels fully as, for example, seen for intracortical veins in Fig. 3A, the low-intensity regions of approximate vertical orientation seen in these EPI images (Fig. S6) showed partial correspondence to regions that were excluded from the orientation preference maps due to their low goodness-of-fit statistics. Thus, one can speculate that intracortical blood vessels may partially be responsible for the regions that were excluded from the laminar orientation preference maps. However, no consistent relationship was evident with columnar orientation preference maps. A single slice examined with higher resolution GRE anatomical images before and after contrast injection, where blood vessels can be visualized more clearly as in Fig 3A and Fig. S2, and arteries can be distinguished from veins (Bolan et al., 2006; Moon et al., 2013), also indicated that a consistent relationship among vessel locations and orientation preference columns did not exist (data not shown), in agreement with previous wCBV fMRI and optical imaging studies (Moon et al., 2013; O’Herron et al., 2016, 2012).

To quantify the degree of columnar organization for preferred orientation, in four cats we calculated the absolute angular deviation (see Materials and Methods) which indicates the similarity that adjacent voxels across cortical depth will have the same preferred orientation. The mean of the absolute value of this angular deviation were 18.1°, 16.9°, 16.1°, and 16.3° for four individual cats and was 16.8° when averaged across data from all cats (Fig. S7) — which is consistent with the angular deviation of iso-orientation domains of layer 2/3 cat visual cortex measured using single-cell resolution two-photon imaging (Ohki et al., 2006).

To further confirm the existence of the columnar organization that we observed in the wCBV fMRI data obtained with axial slices (Fig. 3), we also examined orientation maps from data collected using sagittal slices (*n* = 3 cats). In the sagittal plane, iso-orientation preference across cortical layers can be readily observed in a restricted region of the cortex without having to use cortical flattening (Fig. S8). Having revealed the columnar organization for orientation preference with fMRI for the first time, we next examined how orientation selectivity (the sharpness of tuning) changes across cortical layers.

### 3.4. Depth-dependent orientation tuning from wCBV fMRI

**Fig. 4.**
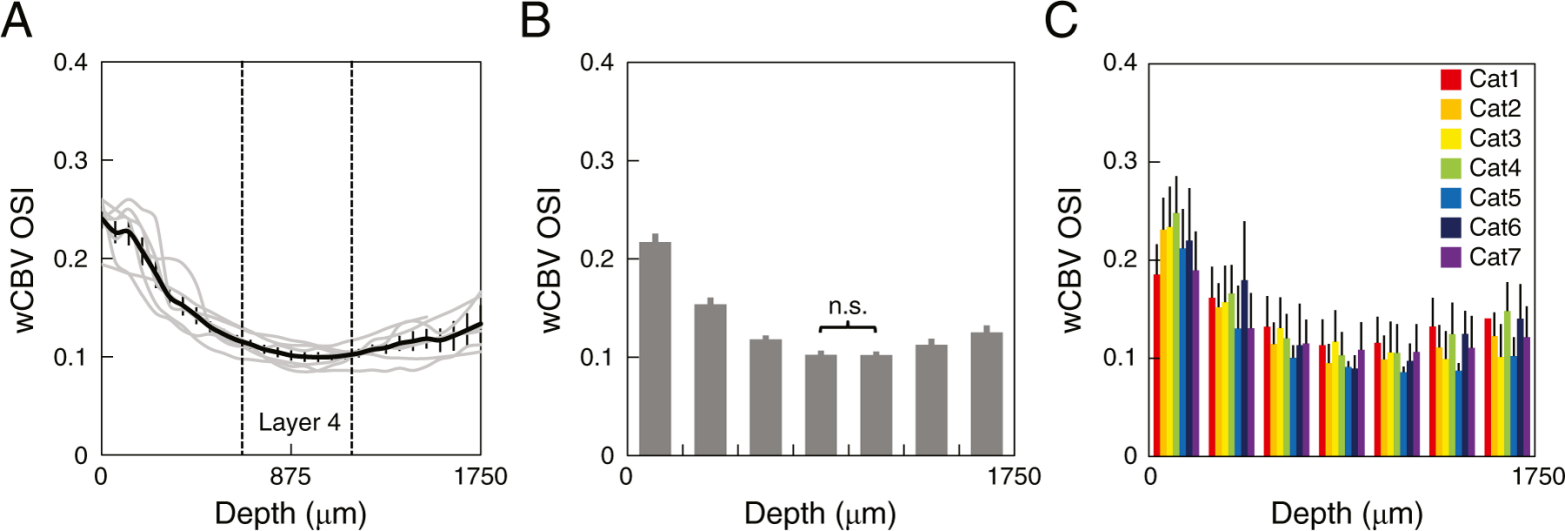
Depth-dependent orientation selectivity index (OSI) estimated from wCBV fMRI. (A) Depth-dependent OSI calculated from stimulus-induced wCBV signal changes. Data from individual cats shown as gray lines, mean from 7 cats shown as black line and error bars are ±1 SEM. The spatial location of layer 4 is indicated by the dashed vertical lines. (B) Average OSI values (binned in 250 *µ*m steps); the differences between the binned OSI values were statistically significant except for the depth between 750 and 1000 *µ*m versus the 1000–1250 *µ*m bins. (ANOVA *P* < 0.05 for each pair of consecutive 250 *µ*m bins). All analyzed voxels had a R^2^ > 0.2 for orientation tuning curve fits. (C) Depth-dependent OSIs of wCBV fMRI where bars represent the individual cats’ OSI calculated from the equal-distanced depth aligned voxels binned into 250 *µ*m thick slabs.

Orientation selectivity index (OSI) (see Materials and Methods) was calculated from the flattened wCBV fMRI data acquired with axial slices and directly from the data obtained with sagittal slices. These calculations were performed for each individual cat and subsequently averaged across 7 cats (Fig. 4). Because the imaging data were interpolated to a grid with 62.5 *µ*m resolution during the flattening and the layerification process, the OSI appears to change smoothly across cortical depth (Fig. 4A). Choosing a cortical laminar-relevant binning (250 *µ*m) across cortical depth, OSI still progressively decreased from the surface into cortical layer 4 (Fig. 4B), the overall OSI ranged between 0.09 and 0.27 across cortical depth, and the group averaged OSI across all depths was 0.14 ± 0.01 (±1 SEM) (*n* = 7) (Fig. 4). The weakest selectivity was obtained at 950 *µ*m (OSI = 0.12), corresponding to the middle of layer 4.

### 3.5. Orientation tuning of single vessel responses by multi-photon optical imaging

**Fig. 5.**
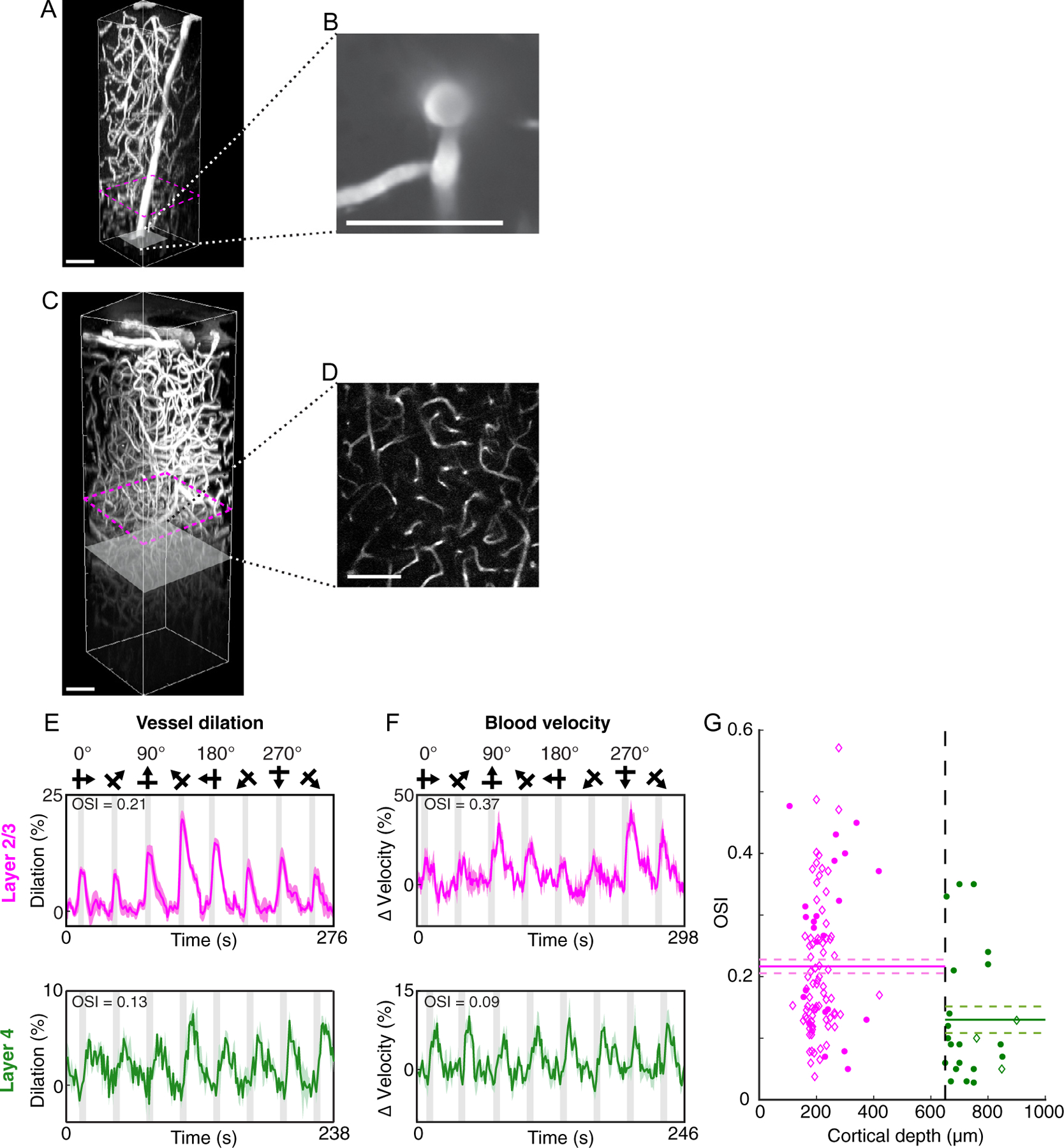
Orientation selectivity of single-vessel dilation and blood velocity in cortical layers 2/3 versus layer 4 using optical imaging. (A–D) Anatomy of the vasculature from visual cortical layers 1 through 4 using either two-photon or three-photon imaging. (A) Side view perspective of a 3D volume imaged using two-photon microscopy where the blood vessels were labeled with Alexa 680 dextran. The maximum imaging depth shown was 850 *µ*m below the brain surface. The gray slice corresponds to the single z plane shown in panel B. (B) Dorsal view of a single z plane at 850 *µ*m depth, imaged at a higher zoom compared to the data shown in panel A. (C) Side view perspective of a 3D volume from another cat using three-photon microscopy and where blood vessels were labeled with fluorescein dextran. The maximum imaging depth with three-photon imaging was 1200 *µ*m. The gray slice corresponds to the single z plane image shown in panel D. (D) Dorsal view of a single z plane at 830 *µ*m depth. Magenta dashed lines in panels A and C indicate the upper limit of layer 4 (650 *µ*m). All scale bars shown in A-D are 100 *µ*m. (E) Time courses of dilation responses of two individual arterioles to visual stimulation (top panel shown in magenta: layer 2/3, depth 234 *µ*m, 6 trials); bottom panel shown in green: layer 4, depth 900 *µ*m, 3 trials). Solid lines and error bands represent the mean and SEM dilation responses; gray vertical bars represent the periods of visual stimulation. (F) Time courses of changes in blood velocity upon visual stimulation in two individual blood vessels (top panel shown in magenta: layer 2/3, 418 *µ*m depth, 3 trials; bottom panel shown in green: layer 4, depth 700 *µ*m, 4 trials). The OSI calculated from responses in each of the 4 example vessels are shown above the time course plots. (G) All OSI values measured for dilation (diamonds) and velocity (circles) from individual layer 2/3 (magenta) and layer 4 (green) vessels across cortical depth. Solid lines represent the mean and dashed lines represent the SEM. The vertical dashed line in black delineates the superficial boundary of layer 4 at 650 *µ*m.

We next used two-photon and three-photon imaging to measure orientation selectivity in individual blood vessels to determine if high-resolution fMRI data could match single-vessel hemodynamic responses. Unlike the case in a “thin” mouse visual cortex, multi-photon imaging in the “thick” cat visual cortex can only be performed from the brain surface through cortical layer 4 (Fig. 5A–D). With the current technology, imaging cortical layers 5 and 6 in cats is not feasible. After labeling blood plasma with fluorescent dyes, we measured artery dilation and blood velocity in layers 2/3 and layer 4 of cat area 18 to drifting grating stimuli and found that all vessels showed visually-evoked responses (Fig. 5E–G). Qualitative examination of the time courses of responses for dilation and blood velocity showed similar trends across cortical layers (compare panels in Fig. 5E and F). More specifically, layer 4 responses appeared to be relatively less tuned for stimulus orientation whereas layer 2/3 responses demonstrated higher selectivity. We quantified single-vessel OSI across all blood vessels (Fig. 5C). In the population of blood vessels we examined, single vessel OSI was significantly lower in layer 4 compared to layer 2/3 (*P* < 0.002, Welch’s *t*-test; layer 4 OSI = 0.13 ± 0.02, *n* = 23 vessels mean ± SEM and layer 2/3 OSI = 0.22 ± 0.01 mean ± SEM, *n* = 103 vessels).

We then determined if the two different measures (dilation and blood velocity) were equivalently matched in reflecting laminar changes in orientation selectivity. Optical imaging contrast deep in the cat visual cortex, i.e., > 650 *µ*m below the pial surface, is lower than in superficial layers. As a consequence of this limitation, at > 650 *µ*m depth, blood velocity measurements are faster and easier to obtain than dilation measurements because they are generated from line scans rather than frame scans. Hence, our layer 4 sample had many more vessels where blood velocity was examined (compared to dilation measurements), but this has no bearing on the endogenous distribution of arteries and capillaries across cortical layers (see Table 1 below). Nevertheless, within cortical layer 2/3, consistent with the qualitative examination of the time courses of response (see Fig. 5E,F), the population OSI between dilation and blood velocity was indistinguishable (*P* = 0.08 Welch’s *t*-test; see Fig. S10A for individual data points and statistics). In comparing layer 2/3 and layer 4 selectivity, in addition to the pooled data shown in Fig. 5G, we had sufficient statistical power for comparing just the blood velocity data across cortical layers. Consistent with the qualitative time course responses shown in Fig. 5E,F, the population OSI for blood velocity in layer 4 was significantly weaker than in layer 2/3 (*P* < 0.005, Welch’s *t*-test, see Fig. S10B for individual data points and statistics). Thus, there was a consistent reduction of OSI from layer 2/3 to layer 4, regardless of whether vessel dilation and blood velocity data were grouped together or whether velocity data were analyzed separately.

### 3.6. OSI comparison between wCBV fMRI voxels and single-vessel optical imaging data

**Fig. 6.**
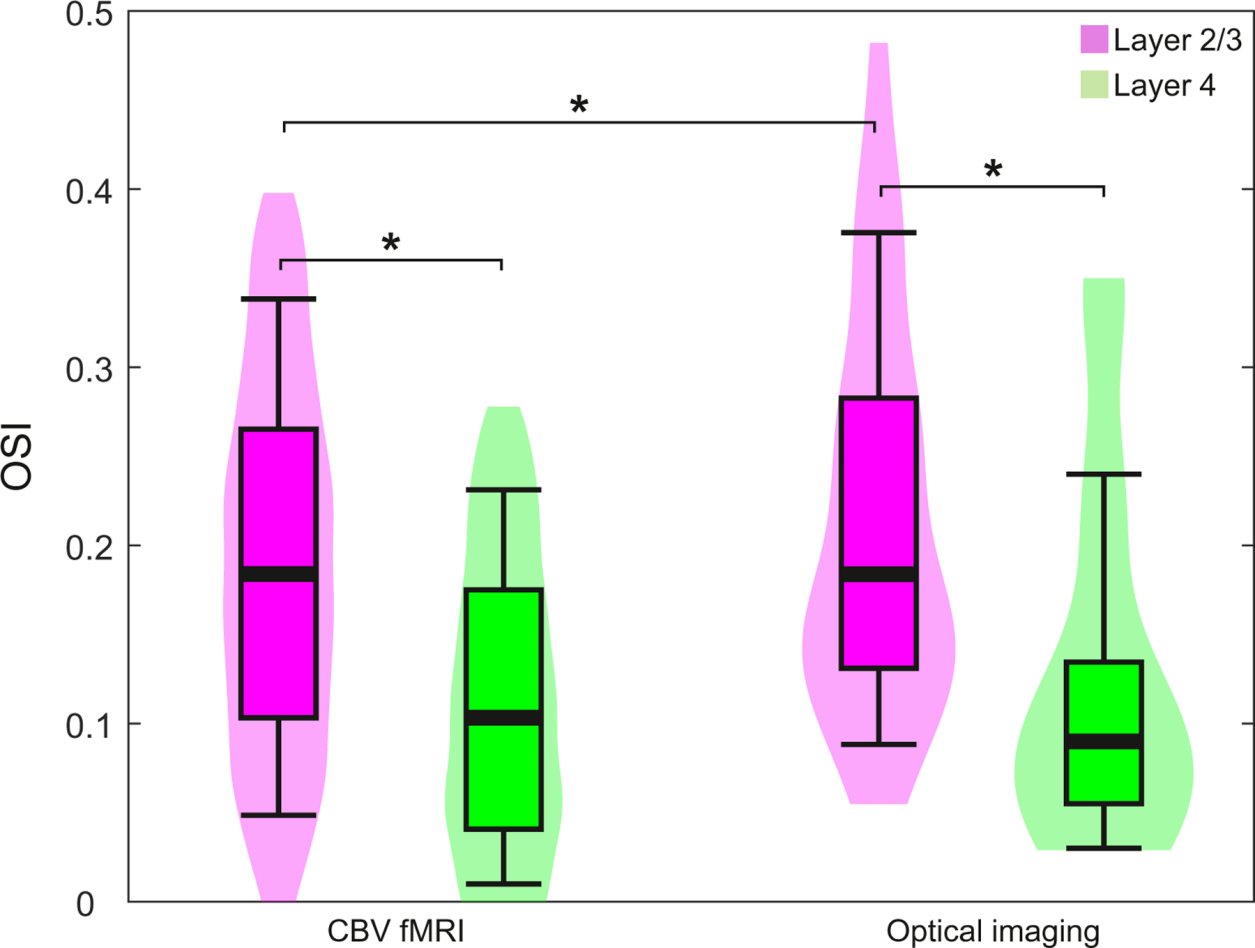
Orientation selectivity index (OSI) obtained from wCBV fMRI and optical imaging in layers 2/3 and 4. The box-violin plots indicate the OSIs in layer 2/3 (magenta) and layer 4 (green). In each box, bold horizontal lines represent the median, boxes the 25% and 75% quartiles and whiskers the 95% confidence intervals. The shaded violin represents the overall distribution of the data where the width indicates the frequency and the height represents the minimum and maximum values. The cortical depth sampled in optical imaging was 160–410 *µ*m for layer 2/3 and 650–910 *µ*m for layer 4. wCBV fMRI data was interpolated to approximately match the same depth. Asterisks indicate significant OSI differences at *P* < 0.05 (Welch’s *t*-test).

Orientation selectivity in cortical layer 4 was found to be lower than in layers 2/3 in both wCBV fMRI (Fig. 6) and multi-photon imaging of single-vessel hemodynamic responses (Fig. 6B). However, because fMRI and single-vessel imaging sample hemodynamic responses at largely different spatial scales, the OSI measured by the two methods could be different. Therefore, we directly compared the OSI measured by the two imaging modalities and found that OSI obtained from the two methods were nearly identical and showed very similar differences between layers 2/3 and layer 4 (Fig. 6). The OSI data from both modalities were averaged for the depths spanned by the single vessel measurements in layer 2/3 (160–410 *µ*m depth) and in layer 4 (650–910 *µ*m depth) and across subjects, and plotted separately for the comparison (Fig. 6). These depth ranges are not the full span of layer 2/3 and layer 4 but only the regions within these layers where individual vessel physiology data were obtained. The full span of layer 2/3 and layer 4 were defined histologically (see Figs. 2B,C and 5G). Most importantly, with both imaging modalities, the average OSI in layers 2/3 was significantly higher than in layer 4 (fMRI: layer 2/3 0.186 ± 0.014, layer 4 0.109 ± 0.010, *P* = 0.001; optical imaging: layer 2/3 0.220 ± 0.01, layer 4 0.130 ± 0.02, *P* = 0.001; mean ± SEM in all cases).

**Fig. 7.**
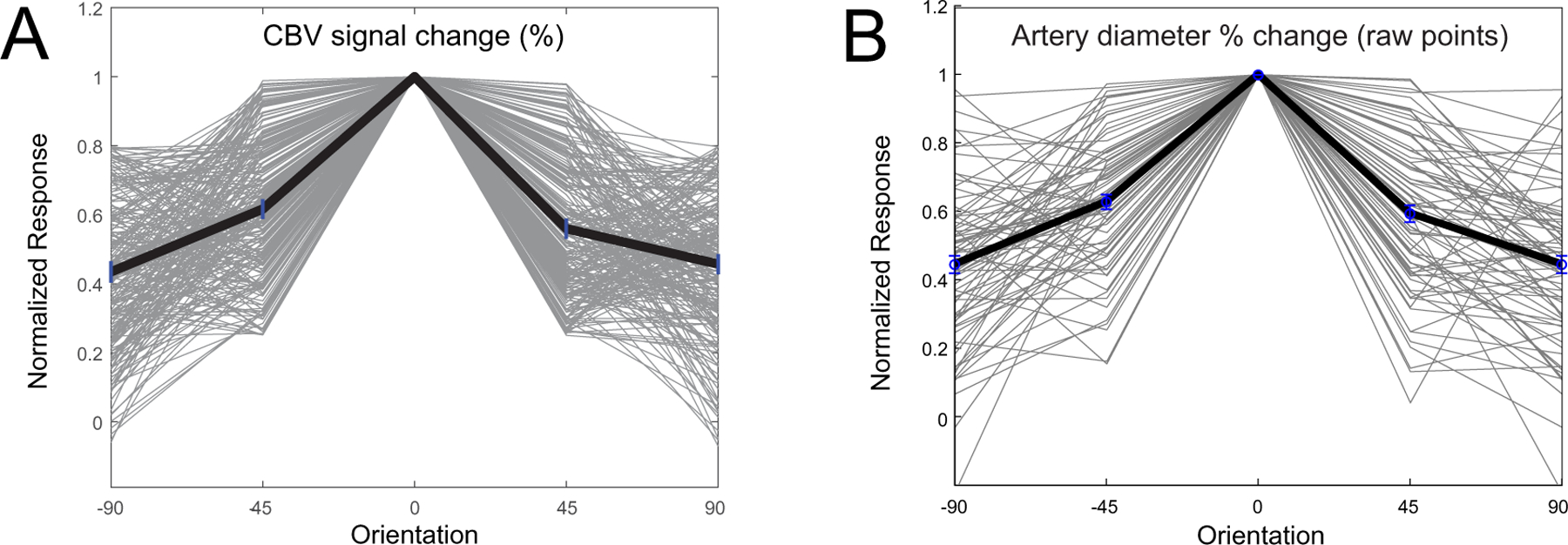
Comparison of cortical layer 2/3 tuning curves between wCBV fMRI (panel A) and artery dilation from optical imaging (B). The CBV data are for all voxels taken from cat 1. Refer to Fig. 4C for the remarkable consistency of OSI measurements across all seven cats where fMRI was performed. In order to compare all tuning curves, for each voxel or blood vessel, the peak response was re-centered to the zero-degree orientation. The tuning curves between fMRI and optical imaging are very similar, as is confirmed by the OSI measurements (CBV layer 2/3 OSI = 0.19 ± 0.09 mean ± SEM, *n* = 300 voxels vs. artery dilation OSI 0.21 ± 0.01 mean ± SEM, *n* = 80 vessels). Also note the similarity of the tails of the tuning curves Y axis ∼ 0.4 across both imaging modalities.

While there is an established literature on the dependence of wCBV fMRI on artery dilation (He et al., 2018; Nagaoka et al., 2006; Smirnakis et al., 2007; Yacoub et al., 2006; Zhao et al., 2007), these metrics have never before been compared in terms of feature selectivity in the parenchyma. Thus, to more precisely determine the consistency of orientation selectivity across wCBV fMRI and artery dilation measurements, we compared the tuning curves for these two measures in cortical layer 2/3 (Fig. 7). The orientation tuning curves across voxels and vessels were remarkably similar in layer 2/3 such that even the tails of the tuning curves matched (compare panels A and B in Fig. 7). Moreover, the OSI was different by only 10% between wCBV fMRI and artery dilation (CBV OSI = 0.19 ± 0.09 mean ± SEM and artery dilation OSI = 0.21 ± 0.01 mean ± SEM). The main focus of our study was on laminar differences, and when comparing across layer 2/3 vs. layer 4 within any single imaging modality, differences in OSI were ∼ 50–60% (see Figs. 5–6 and S8).

## 4. Discussion

fMRI is currently the most extensively used technique for investigating human brain function. Yet, most studies use millimeter or larger spatial resolution, unable to account for the critical roles played by different layers of the neocortex. Due to recent improvements in the sensitivity and specificity provided by ultrahigh magnetic fields (Ugurbil, 2014, 2018), interest has grown in using high resolution fMRI for studies with laminar differentiation in both animal and human studies. To date, such high resolution studies have only shown that laminar differences in fMRI signals are detectable and, in some case, show alterations linked to a task (reviews: Lawrence et al., 2019; Norris and Polimeni, 2019; Poplawsky et al., 2019; Weldon and Olman, 2021). However, correlates of neuronal feature selectivity, for example orientation selectivity, across cortical layers has not been demonstrated. Here we show that high-resolution wCBV fMRI can detect a largely columnar organization of preferred orientation and laminar differences in orientation selectivity index; furthermore, we demonstrate that these laminar differences are near identically represented in hemodynamic signals captured from individual blood vessels via optical imaging (Fig. 6).

### 4.1. Columnar organization of orientation preference based on hemodynamic imaging

Our wCBV fMRI results show a largely columnar organization of orientation preference, both in the qualitative examination of orientation maps and via our quantification of the degree of columnar-likeness, i.e., angular deviation. Thus, our fMRI results are similar to previous single-unit electrophysiological studies which include data from all cortical layers (Murphy and Sillito, 1986; Shmuel and Grinvald, 1996). Irregularities in map precision in cat visual cortex (tilting of individual domains away from tangential to the cortical surface and interruptions or breaks in continuity of the map) are evident in multiple imaging modalities and in single-unit recordings. Aside from the fMRI data shown here, such irregularities have also been seen in functional optical coherence tomography imaging of V1 (Nakamichi et al., 2018) and in the two single-unit studies cited above. Until we have three-photon neuronal map data from cortical layers 4, 5 and 6 along with improved fMRI techniques, all the potential sources of these imperfections in orientation preference maps will remain unknown. Three possible contributing factors are as follows: First, hemodynamic signals generally have less orientation selectivity than synaptic and spiking neural activity in cat visual cortex (O’Herron et al., 2016). Second, layer 4 signals in particular are less orientation-selective than signals from other cortical layers. This applies for fMRI and single-vessel hemodynamic signals in cat visual cortex (the current study) and neural signals, thus far, only measured in other species using single-unit recordings (Ringach et al., 2002; Van Hooser et al., 2013). Third, the sampling radius of detecting single-unit activity from large neurons and local field potentials (LFPs) measured by extracellular metal electrodes can sometimes span hundreds of micrometers (Berens et al., 2008; Logothetis, 2008; Olshausen and Field, 2005; Xing et al., 2009). It has long been established that every part of the neuronal membrane is capable of generating action potentials (Buzsaki, 2004; Henze et al., 2000; Llinás, 1988). Surrogates of synaptic activity are relevant because our previous work in cat visual cortex showed that OSIs derived from synaptic responses (imaged precisely on the scale of micrometers) are more closely matched to single-vessel hemodynamic responses, as compared to the OSIs derived from spiking responses (O’Herron et al., 2016). Moreover, also in the cat visual cortex, the potentially large sampling radius of single-unit electrodes may have led to an initial incorrect conclusion regarding the spatial organization of cells near orientation pinwheel singularities. Specifically, orientation pinwheel singularities were reported to have a “salt-and-pepper” like mixture of neurons with different orientation preferences but all sharply-tuned (Maldonado et al., 1997), however, subsequent multi-photon imaging experiments in the cat visual cortex showed otherwise (O’Herron et al., 2012; Ohki et al., 2006).

### 4.2. Cortical location, efficacy and selectivity of thalamic inputs explain laminar changes in OSI when using hemodynamic signals

In neurons, spike threshold dramatically enhances feature selectivity. Specifically, orientation and direction selectivity improve 50–100% from single neuron computation-synaptic input to spiking output via spike threshold (Carandini and Ferster, 2000; O’Herron et al., 2016; Priebe and Ferster, 2008, 2012). Blood vessels do not have this luxury of a threshold computation in their dilation signaling and mechanics (Hillman, 2014; Iadecola, 2017). Layer 4 neurons in V1 receive three distinct classes of inputs: (a) from the visual thalamus, i.e., the lateral geniculate nucleus (LGN), (b) from other layer 4 neurons, and (c) from layer 6 pyramidal neurons (Stratford et al., 1996). Of all the classes of synapses in V1, the thalamic inputs are twice as strong as layer 4 inputs and eight times stronger than layer 6 inputs (Stratford et al., 1996). Critically, in cat visual cortex, thalamic inputs are completely untuned to stimulus orientation (Ferster et al., 1996; Kara et al., 2002, 2000; Reid and Alonso, 1995). Thus, the dominant synaptic input in layer 4 is untuned to stimulus orientation and this should be reflected in the dilation responses of individual blood vessels. In agreement with this prediction, we indeed show a significantly lower OSI in layer 4, as measured with optical imaging at the level of single blood vessels and with wCBV fMRI.

### 4.3. The spatial resolution of wCBV fMRI that matches single-vessel hemodynamic responses

Two minimum conditions are necessary for fMRI signals to match single-vessel hemodynamic responses. First, the spatial resolution needs to be fine enough so that individual voxels can be correlated into known cortical layers. Second, the voxel resolution of fMRI needs to be finer than the point spread function (PSF) of hemodynamic responses. In cat V1, we previously showed that blood flow changes induced by orientation selective stimuli has a tangential PSF of 470 *µ*m at Full Width Half Maximum (FWFM) (Duong et al., 2001). We have also demonstrated previously that single vessel dilation spreads from one cortical column into adjacent columns by more than 600 *µ*m, resulting in the orientation selectivity of individual vessels being less than the orientation selectivity in neighboring neurons (O’Herron et al., 2016). Thus, by selecting a wCBV fMRI voxel size of 250 *µ*m, both of the above-mentioned conditions were met. In different cortical regions or species, the sensory feature of interest might be wider or narrower than orientation selectivity in cat V1 and thus a different fMRI voxel resolution may be needed to capture laminar differences in feature selectivity (Denfield et al., 2016).

### 4.4. wCBV fMRI changes across cortical layers-significance for other fMRI techniques

wCBV fMRI detects blood volume changes because of the exogenous intravascular contrast agent used. However, the approach is not applicable to humans due to safety concerns with the exogenous contrast agent. Alternative methods of functional mapping suitable for human use are Blood-oxygen-level-dependent (BOLD) fMRI (Ogawa et al., 1992), cerebral blood flow (CBF) based fMRI (Detre et al., 1992; Duong et al., 2001; Kwong et al., 1992), and Vascular-Space-Occupancy (VASO) method (Huber et al., 2018; Lu et al., 2013). VASO is thought to reflect CBV changes; as such, it should in principle also be able to detect laminar differences in OSI, consistent with our present results. Similarly, when executed so that CBF reflects tissue perfusion (i.e., capillary flow and exchange with tissue), CBF fMRI has been shown to be highly specific (Duong et al., 2001, 2000), and thus may also show laminar variations in OSI. However, BOLD signals are more complicated. BOLD reflects the deoxyhemoglobin (dHb) content, which is altered as a consequence of stimulus-induced blood flow and oxygen consumption rate changes. This dHb perturbation does not remain localized; rather, it propagates through large veins, persisting possibly as much as a few millimeters in the human brain before it is diluted out (Turner, 2002). This obfuscates the spatial precision of GRE BOLD fMRI (Ugurbil, 2016), the most commonly employed BOLD fMRI approach. It is possible to suppress this confound by using BOLD contrast based on spin-echoes (SE BOLD), which restricts the detection of stimulus-evoked dHb alterations largely to dHb containing microvasculature (capillaries and post capillary venules with diameters ≲20 *µ*m) when executed properly so that SE condition is fulfilled as closely as possible (Ugurbil, 2016); as such, SE BOLD fMRI has higher specificity to neuronal activity (Ugurbil, 2016; Yacoub et al., 2008, 2007) and should be able to measure laminar variations in OSI. However, VASO, perfusion fMRI and SE BOLD fMRI all suffer low SNR, which is a major limitation for very high-resolution fMRI. Recently introduced fMRI denoising techniques (Vizioli et al., 2021) and/or use of magnetic fields higher than what has been available to date are expected to significantly alleviate this limitation, enabling layer-specific selectivity measurements like we performed in cats to also be made in humans.

## 5. Conclusions

Our results show that wCBV fMRI signals can capture critical aspects of cortical laminar function. This requires careful selection of voxel resolution that is finer than the major cortical layers and smaller than the lateral point spread of single-vessel responses with adequate SNR. However, because hemodynamic signals will typically be less selective than neural responses, capturing laminar differences in neuronal responses will still require critical new advances. Such advances will likely include improvements in the sensitivity of fMRI methods (Vizioli et al., 2021), imaging of synaptic and spiking responses across cortical layers (particularly layers 4–6) with 3-photon imaging to obtain the necessary neural maps (Liu et al., 2020; Yildirim et al., 2019), and neural-to-vascular coupling models in order to map features of neural activity into predictions of activity observed for individual fMRI voxels (Allen et al., 2021; Havlicek and Uludag, 2020).

## Supplementary Material

1

## Figures and Tables

**Table 1:** Baseline diameters of vessels sampled with optical imaging.

Category	Baseline vessel diameter range (*μ*m)	Baseline vessel diameter (Mean ± SEM)	Number of vessels (N)
ayer 2/3 dilation	8–40	16.4 ± 5.5	80
Layer 2/3 blood velocity	3.6–10.5	6.5 ± 2.2	23
Layer 4 dilation	25–40	31.7 ± 7.6	3
Layer 4 blood velocity	4.8–13.9	9.6 ± 2.9	20

## Data Availability

fMRI and optical imaging data may be shared with other research groups pending a formal data sharing agreement. For optical imaging data, as noted in the Methods, the algorithm we developed for assaying blood flow in optical imaging data has been published previously, e.g., see https://www.mathworks.com/matlabcentral/fileexchange/42019-hybridvel. For fMRI data, as noted in the Methods, Analysis of Functional Neuroimages (AFNI) software was used.
