# Supplementary material for: Cortical layer-specific differences in stimulus selectivity revealed with high-field fMRI and single-vessel resolution optical imaging of the primary visual cortex
Source: Neuroimage. Author manuscript; Available in PMC 2022 May 1. (PMC9048976; doi:10.1016/j.neuroimage.2022.118978)
Supplement: 1 [file NIHMS1792865-supplement-1.docx]

**SUPPLEMENTARY INFORMATION**

**Fig. S1.** **Removal of large vein activation in the F-stat map of wCBV signal change.**

(A) Anatomical GRE image shows a representative coronal slice of area 18 (left) depicting the presence of deoxyhemoglobin containing blood vessels as dark lines. (B) Image of the logarithm of the standard deviation divided by the mean signal intensity (log(Std/Mean) of the wCBV fMRI time series for the same slice as (A). (C) Histogram of voxel-wise log(Std/Mean); the red dashed line shows the cut-off threshold log(Std/Mean) of 1.6 that can be used to remove spurious "activation" adjacent to pial surfaces (see Materials and Methods). (D) The voxel-wise image mask generated using the cut-off threshold log(Std/Mean) of 1.6; the voxel in the bright pixel locations were excluded from subsequent analyses to generate maps of stimulus-induced responses. (E) Two functional maps on the coronal plane superimposed on a GRE image before (left) and after-applying a threshold to eliminate voxels with log(Std/Mean) > 1.6 from the functional map. The scale bar denotes 1 mm.


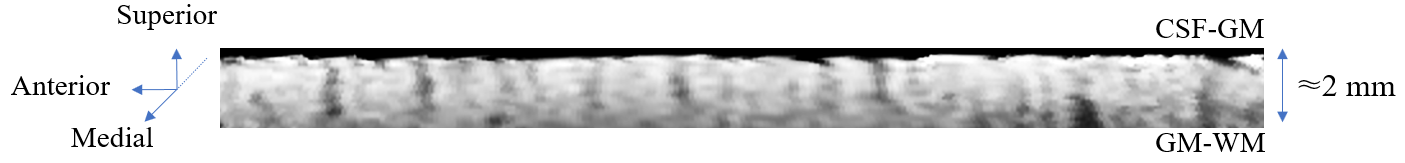


**Fig. S2. Example of a flattened cortex derived from GRE anatomical images obtained before applications of the exogenous contrast agent.**

The dark lines represent the intracortical emerging veins that run perpendicular to the cortical surface; they are dark because of the presence of deoxyhemoglobin in them (i.e. The BOLD effect). In the flattened image, this perpendicular orientation to the cortical surface is maintained and they appear to run vertically. This gives confidence in the flattening algorithm.

**Fig. S3.** **Group-averaged depth profile of wCBV signal change (averaged across 7 animals).** Zero depth indicating the cortical surface. The maximum activation was found near 950 μm cortical depth in area 18, corresponding to layer 4. Activation increased up to layer 4 and gradually decreased in deeper cortical depths. Asterisks indicate the significant difference in activation between cortical depth (ANOVA, *P <* 0 .001*)*.

**Fig. S4. Functional organization of the orientation preference map across the cortical surface using wCBV fMRI.** (A) The Poincaré-Hopf index map ([Brasselet et al., 2009](#_ENREF_1)) calculated from a flattened cortex represents the circular variation of orientation preferences around a voxel; high value (red) indicates the preferred orientation rapidly changing around a singular point and *vice versa* for the low value (blue). (B) The Poincaré-Hopf index map on a single plane at 250 μm cortical depth; circles (zoomed-in image) indicate the location of detected pinwheel centers with black lines displaying the straight-line distances between neighboring pinwheel centers, calculated by Delaunay triangulation (see details in Methods and Materials). (C) The orientation map estimated from the flattened cortex shown in panel B; colors corresponding to different orientation preferences and black lines indicate boundaries of hyper-column defined by Voronoi tessellation ([Fortune, 1995](#_ENREF_2)). Zoomed-in images in panel (C) demonstrate the existence of clockwise (CW) and counter-clockwise (CCW) pinwheels. The average spacing between pinwheel centers detected and calculated by Poincaré-Hopf index and Delaunay triangulation was 0.83±0.5 mm (±1 SEM) in the 3D volume covered by the imaging slices, considering distances both parallel to cortical surface (within layer) and across cortical layers, consistent with a previous report ([Mazade and Alonso, 2017](#_ENREF_3)).

**Fig. S5. R^2^ statistics of the von Mises** **tuning curve fitting.**

(A) Histogram of R^2^ values obtained in the von Mises tuning curve fitting for flattened A18 area (*n* = 588 voxels from 7 cats). The average R^2^ is 0.31. (B) Average R^2^ values across cortical depth, showing individual cats (gray lines) and averaged data (black line). (C) Average R^2^ values across preferred orientation in voxels presented as individual animals (gray lines) and group-averaged data (black line).

**
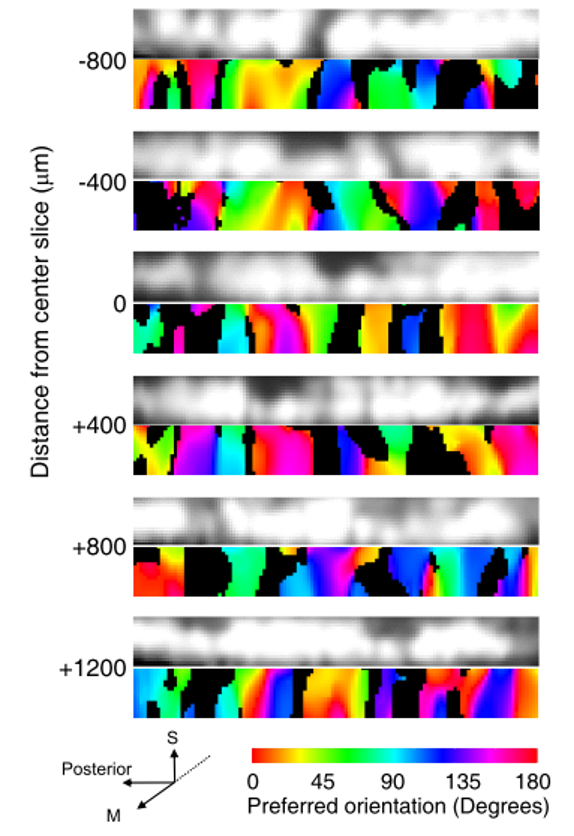
**

**Fig. S6:** **The flattened EPI slices (after contrast agent infusion) across the cortical thickness and the corresponding laminar orientation preference maps derived from them**. The orientation preference maps are the same as six of the slices presented in Fig. 3C. The penetrating intracortical blood vessels, both arterial and venous, can appear in these EPI slices as dark vertical stripes due to signal loss associated with them, arising from the contrast agent induced magnetic susceptibility difference between the blood vessel interior and the surrounding tissue. The mask-out regions in that orientation maps that are shown in solid black have low goodness of fit (the goodness-of-fit of orientation tuning curve < 0.2) (See Fig. 3). These mask-out regions appear to partially overlap regions showing vertically aligned dark “stripes” in the EPI images.

**Fig. S7. Angular deviation of orientation preference in columns.**

The deviations of orientation preferences (°) within columns from a flattened area 18 (number of columns, *n* = 12436~13228 depending on an animal, 4 animals). (A) Shows the representative sagittal column map (left-right for causal-rostral), which was constructed from the flattened cortex, derived from the axially acquired images. The voxel column is indicated by the dashed black lines in the zoomed image. In order to compare with the control condition, we generated scrambled map by shuffling the location voxels (right panel). (B) The mean deviations were plotted for four subjects individually (cat 1-4), which were 18.1°, 16.9°, 16.1°, and 16.3° (std., 9.8, 9.9, 9.7, and 10.1 respectively), indicating the angular deviation was 16.8±0.44° on average (*n* = 4, S.E.M). The mean deviation was significantly lower (*P* < 0.0001) than the mean deviation of control condition (31.56 ± 0.50°, S.E.M). (C) shows the histogram of polled angular deviation for columns (4 animals). The solid and dotted line indicates the mean and std. of angular deviation respectively.

**Fig. S8.** **Columnar organization of orientation preference evident in sagittal plane fMRI imaging.** Sagittal CBV fMRI acquisition from a single slice in a single cat shows a columnar organization of the orientation preference map, equivalent to data derived from axial slices shown in Fig. 3. The zoomed-in view shows a subset of orientation domains.

**Fig. S9. Equivalent labeling of cortical layer 4 with two different VGLUT2 antibodies.**

(A) Coronal section from cat area 18 labeled with Millipore AB2251-I. (B) Coronal section from cat area 18 labeled with Synaptic Systems 135403. Scale bars are 200 μm.

**Fig. S10. Further comparisons of optical imaging data within and across cortical layers.**

(A) Within layer 2/3, the small OSI difference between blood velocity and vessel dilation was not significantly different, i.e., velocity OSI = 0.26 ± 0.03 mean ± SEM, n = 23 vessels vs. dilation OSI = 0.21 ± 0.01 mean ± SEM, n = 80 vessels; *P* = 0.08, Welch's *t*-test. (B) From layer 2/3 to layer 4, OSI measured from blood velocity was significantly reduced (denoted by the asterisk), i.e., layer 2/3 OSI = 0.26 ± 0.03 mean ± SEM, n = 23 vessels vs. layer 4 OSI = 0.14 ± 0.02 mean ± SEM, n = 20 vessels; *P* < 0.005, Welch's *t*-test. Error bars in panels A and B represent SEM. See Fig. 5 and main text for additional details.

**SUPPLEMENTARY REFERENCES**

Brasselet, J.-P., Seade, J., Suwa, T., 2009. Vector fields on singular varieties. Springer Science & Business Media.

Fortune, S., 1995. Voronoi diagrams and Delaunay triangulations. Computing in Euclidean geometry, 225-265.

Mazade, R., Alonso, J.M., 2017. Thalamocortical processing in vision. Visual neuroscience 34, E007.
